# Maternal obesity and resistance to breast cancer treatments among offspring: Link to gut dysbiosis

**DOI:** 10.1002/cnr2.1752

**Published:** 2022-11-21

**Authors:** Fabia de Oliveira Andrade, Vivek Verma, Leena Hilakivi‐Clarke

**Affiliations:** ^1^ The Hormel Institute, University of Minnesota Austin Minnesota USA

**Keywords:** gut microbiome, immunotherapy, maternal obesity, offspring, short chain fatty acids

## Abstract

**Background:**

About 50 000 new cases of cancer in the United States are attributed to obesity. The adverse effects of obesity on breast cancer may be most profound when affecting the early development; that is, in the womb of a pregnant obese mother. Maternal obesity has several long‐lasting adverse health effects on the offspring, including increasing offspring's breast cancer risk and mortality. Gut microbiota is a player in obesity as well as may impact breast carcinogenesis. Gut microbiota is established early in life and the microbial composition of an infant's gut becomes permanently dysregulated because of maternal obesity. Metabolites from the microbiota, especially short chain fatty acids (SCFAs), play a critical role in mediating the effect of gut bacteria on multiple biological functions, such as immune system, including tumor immune responses.

**Recent Findings:**

Maternal obesity can pre‐program daughter's breast cancer to be more aggressive, less responsive to treatments and consequently more likely to cause breast cancer related death. Maternal obesity may also induce poor response to immune checkpoint inhibitor (ICB) therapy through increased abundance of inflammation associated microbiome and decreased abundance of bacteria that are linked to production of SCFAs. Dietary interventions that increase the abundance of bacteria producing SCFAs potentially reverses offspring's resistance to breast cancer therapy.

**Conclusion:**

Since immunotherapies have emerged as highly effective treatments for many cancers, albeit there is an urgent need to enlarge the patient population who will be responsive to these treatments. One of the factors which may cause ICB refractoriness could be maternal obesity, based on its effects on the microbiota markers of ICB therapy response among the offspring. Since about 40% of children are born to obese mothers in the Western societies, it is important to determine if maternal obesity impairs offspring's response to cancer immunotherapies.

## 1 OBESITY AND BREAST CANCER

Breast cancer is one of the most common malignant cancers in women around the globe, and the leading cause of cancer related deaths in women.^1^ The exact origin of breast cancer remains unknown, although many non‐modifiable and modifiable factors have been identified.^2^, ^3^, ^4^ Most breast cancers are sporadic; only approximately 5%–10% are heritable and caused by a known or unknown germline mutation. Obesity (body mass index, BMI > 30) is a major modifiable risk factors for cancer.^5^ According to Centers for Disease Control and Prevention (CDC), in 2017–2018 the age‐adjusted prevalence of obesity among U.S. adults was 42.4%.^6^ Consequently, 50 000 new cases of cancer in the United States, especially breast cancer, can be attributed to obesity.^7^, ^8^ Obesity is associated with increased risk to develop breast cancer in postmenopausal women,^9^, ^10^, ^11^, ^12^ and triple negative breast cancer (TNBC) in premenopausal women.^13^, ^14^, ^15^ Visceral obesity; that is, accumulation of fat around the visceral organs, reflected as a waist/hip ratio of over ≥0.95,^16^ increases the risk of all types of premenopausal breast cancer.^17^ Obesity is also associated with 35%–40% increased risk of breast cancer recurrence and death regardless of menopausal status at the diagnosis.^18^, ^19^

### 1.1 Maternal obesity and breast cancer risk among the offspring

The adverse effects of obesity on breast cancer risk may be most profound if the excess body weight begins to impact an individual already in the womb through a pregnant obese mother.^20^ To promote a healthy pregnancy and long‐term outcome for the next generation, women's pre‐pregnancy BMI should be between 19 and 28. Being obese before pregnancy reduces fertility and increases the incidence of early miscarriage and adverse pregnancy‐related events, such as gestational diabetes and pre‐eclampsia. Underweight women (BMI < 19) also exhibit impaired fertility and increased incidence of early miscarriage.^21^ The prevalence of overweight and obesity among women of childbearing age in the United States is 55% for non‐Hispanic Caucasian women and over 70% for African‐American women.^22^ These numbers are constantly increasing: from 2016 to 2019 an 11% increase in pre‐pregnancy obesity was observed in the United States.^23^

Findings obtained in human studies indirectly suggest that maternal obesity increases daughter's breast cancer risk. A link between high birthweight and increased breast cancer risk has been found in several studies.^24^, ^25^, ^26^, ^27^ High birth weight and neonatal adiposity are linked to maternal obesity, before and/or during pregnancy, and excessive pregnancy weight gain^28^, ^29^, ^30^, ^31^, ^32^ as well as maternal intake of fat during pregnancy.^33^ In a preclinical animal study, maternal intake of obesity‐inducing diet (OID) during pregnancy increased female offspring's birth weight and caused earlier onset of estrogen receptor positive (ER+) mammary cancer.^34^ We also found that maternal obesity increased growth of TNBC among offspring.^35^

### 1.2 Maternal obesity alters responsiveness to breast cancer treatments among offspring

The possibility that maternal obesity before or during pregnancy may pre‐program daughter's breast cancer to be more aggressive, less responsive to treatments and consequently more likely to cause breast cancer related death has been studied in humans. In one study, high birthweight was linked to increased breast cancer mortality.^36^ Further, a Helsinki Birth Cohort Study found a correlation between maternal weight during pregnancy and poor outcomes of different cancers in descendants.^37^ Additional support between maternal obesity and increased risk of breast cancer deaths among daughters may come from the well‐documented racial difference in breast cancer mortality that is significantly higher in women of African‐American than non‐Hispanic Caucasian women,^38^ especially in patients diagnosed with ER+ breast cancer.^39^, ^40^ Although the incidence of obesity at childbearing age is almost twofold higher among African‐American women than non‐Hispanic Caucasian women,^40^, ^41^ it is not clear that maternal obesity causally contributes to higher breast cancer mortality in African‐American women.

The impact of maternal obesity on female offspring's breast cancer mortality has been studied in animal models. Montales et al^42^ investigated in MMTV‐Wnt1‐Tg mice if maternal lard‐based OID altered offspring's response to doxycycline (Dox) chemotherapy. Although the OID exposed offspring exhibited an increased risk of developing mammary tumors, no differences in the response to Dox in the control and OID offspring were noted.^42^ We have investigated in a preclinical rat model if maternal OID affected offspring's response to antiestrogen tamoxifen and the risk of recurrence after therapy was completed. No evidence for an impairment in response to tamoxifen therapy among OID offspring were noted.^35^ However, the risk of recurrence after an equivalent of 5‐years of tamoxifen treatment was completed was threefold higher in the OID exposed offspring than the control offspring.^35^ Thus, human and animal studies suggest that maternal obesity may not only increase daughter's breast cancer risk but also the likelihood of recurrence after therapies have been completed, and hence the fatality from breast cancer is higher in daughters born to obese mothers.

## 2 MECHANISMS LINKING MATERNAL OBESITY TO BREAST CANCER: INFLAMMATION

Several mechanisms have been proposed to mediate the effects of obesity on breast cancer, and they are summarized in reviews by Allott and Hursting,^43^ Smith et al,^5^ Zhao et al^44^ and others. Among the mediating factors are an increase in adipose‐derived estrogens, insulin‐like growth factor 1 (IFG‐1), inflammatory cytokines and chemokines, epigenetic modifications, and changes in various other adipose derived factors, such as leptin and adiponectin. These changes mainly reflect an increase in visceral obesity; that is, visceral adipose tissue (VAT). Obese pregnant women have more VAT than lean pregnant women throughout gestation, and higher VAT to “healthy” subcutaneous fat ratio.^45^ VAT contains many inflammatory and immune cells, and the adipocytes are lipid‐packed and hypertrophic.^46^ An excess accumulation of VAT is linked to increased pro‐inflammatory adipose tissue macrophages (ATMs)^47^ and cytokines,^48^ such as IL6, IL8, and MCP1.^49^ Among the metabolic changes taking place in accumulating hypertrophic adipose cells in VAT is a reduction in adipocyte mitochondrial oxidative capacity; that is, oxidative phosphorylation (OXPHOS).^50^ OXPHOS is a process in which electrons are transported along different complexes in the inner mitochondrial membrane to produce ATP. OXPHOS can be induced when the tricarboxylic acid (TCA) cycle is activated by a reaction generated from fatty acids, amino acids or pyruvate oxidation. Since TCA cycle is a key regulator of cellular metabolism, increase in VAT impairs the ability of cells to maintain optimal energy production to support their function. Savva et al have studied the impact of maternal obesity on metabolism in visceral, subcutaneous and brown adipose tissue in the offspring, and among the many changes, most of which are gender‐specific, an impaired TCA cycle and OXPHOS in female offspring.^51^

Although any of these obesity‐induced changes could explain why obesity increases breast cancer risk and mortality, we will focus in this review on inflammation and immune responses. A causal link between chronic inflammation and cancer is now well accepted.^52^, ^53^ Since obesity induces a low‐grade, chronic inflammation, it has been proposed to explain the obesity‐mediated increased cancer risk and mortality.^54^, ^55^ The impact of maternal obesity on the fetal inflammatory environment and postnatal immune responses has been studied in some detail. Results indicate that increased maternal body weight is linked to inflammation in the mother,^56^ and chronic systemic inflammation^57^, ^58^ and changes in the expression and DNA methylation of immune genes^59^ in the offspring. Changes in immune responses in the offspring born to obese mothers have also been studied. Mice exposed to OID in utero had fewer splenic lymphocytes, thinner thymic cortex and impaired antigen‐specific immune reactions as well as higher levels of TNFα.^58^ We have found in rats that maternal OID impaired antigen presentation and anti‐tumor CD8+ T cell activation in the tumor microenvironment (TME) among the offspring.^35^ In humans, maternal obesity caused reduced monocyte and dendritic cell ex‐vivo response, reduced CD4+ T helper cell numbers, and increased plasma levels of IFNα and IL6 in the umbilical cord blood, compared with offspring of lean mothers.^60^ These findings suggest that maternal obesity impairs T cell activation, and thus consequently perhaps their ability to react to immune checkpoint blockade antibodies.

Consistent with the fact that colonization of the gut microbiome during early life impacts the developing immune system,^61^, ^62^ maternal obesity increases the abundance of inflammation associated microbiome.^63^ In mice, maternal OID modulates the offspring's gut microbiome to increase intestinal expression of IL17A and population of IL17‐producing innate lymphoid cells.^64^ In our study, maternal obesity increased the expression of IL6 and IL17F in the mammary TME in mice and rat offspring.^35^

### 2.1 Obesity and response to immune checkpoint inhibitor therapies

Immune therapies, consisting mainly of immune checkpoint inhibitors (ICBs), have been integrated into the standard of care regiments for advanced melanoma, non‐small cell lung cancer (NSCLC), cutaneous squamous cell carcinoma, urothelial cancer, renal cancer, refractory Hodgkin lymphoma, hepatocellular carcinoma, gastric cancer, and triple‐negative breast cancer (TNBC). The decision to include TNBC to the list of ICB treatable cancers was made in 2019 based on results obtained in a clinical trial, which included 451 untreated metastatic TNBC.^65^ Progression‐free survival was significantly improved among these patients if they received atezolizumab, an anti‐PD‐L1 monoclonal antibody in combination with nanoparticle albumin‐bound (nab)‐paclitaxel^65^ and had tumors that were positive for PD‐L1 expression (≥1%).^66^ ER+ breast cancers are refractory to ICBs as a monotherapy,^67^, ^68^ but treatment with HDAC inhibitors might increase responsiveness.^69^

Less than 30% of cancer patients with cancers identified as potentially ICB responsive benefit from this therapy.^70^ Identifying factors that determine ICB responsiveness or resistance and could thus be targeted to convert refractory patients to be responsive, is an active field of research. It is not known if maternal obesity may program offspring of exhibiting altered ICB response. Paradoxically, adult obesity improves responsiveness to ICB immunotherapy in multiple experimental models and humans.^71^, ^72^ In a study that assessed progression free and overall survival among 250 patients with lung or ovarian cancer or melanoma, ICB therapy was significantly more effective in obese than non‐obese patients.^71^ Other studies have reported similar findings: obese metastatic renal cell carcinoma patients^73^ and obese metastatic melanoma patients^74^ are more responsive to ICBs than non‐obese patients. It also was recently found that obesity might improve TNBC responsiveness toward ICBs in preclinical models.^75^

Understanding the mechanisms by which obesity improves response to ICBs is needed to gain better responsiveness to ICBs among non‐obese patients. One possible, although likely only partial explanation is that adult obesity is linked to upregulation of immune checkpoints such as PD1 on immune cells.^76^, ^77^ The higher PD1 expression could make exhausted CD8+ T cells responsive to ICBs. Another explanation is that obesity improves metabolism in CD8+ T cells,^78^ which in turn invigorates them. Offspring born to obese mothers may not exhibit similar advantage than obese adults when treated with ICBs. Offspring of obese mothers exhibit lower infiltration of CD8+ T cells and higher expression of Treg cells,^35^ and consequently anti‐PD1 therapy is expected to activate mostly immunosuppressive Treg cells. Since half of Western population are born to an overweight or obese mother, when they reach adulthood, these individuals are at an increased breast cancer risk. Hence, it would be important to determine if maternal obesity modifies offspring's responsiveness to ICB.

## 3 GUT MICROBIOTA AND BREAST CANCER

The gut microbiome is the largest immune organ in the human body^79^; however, the importance of gut microbiota for human health has only recently been realized. The reason why the gut microbiota is a critical player in affecting human health is that the gut microbiota has coevolved with humans and interacts with human cells in a manner that is mutually beneficial.^80^ Commensal bacteria in the gut use food supplied by the host but does not feed on host's own tissues. In symbiosis with human cells, the gut microbiota is involved in food digestion and synthesis of the host amino acids, carbohydrates, vitamins and other bioactive compounds.^81^, ^82^ A balanced microbiota also promotes strong mucosal barrier to protect harmful bacteria from leaving the contents of the gut lumen.^83^ Among the most important functions of the gut microbiome is prevention of autoimmune responses, inhibition of inflammation and support for the immune functions.

### 3.1 Gut microbiota

The gut microbiota in humans and rodents is dominated by the phyla Firmicutes and Bacteroidetes (these two compose 90% of the human gut microbiota^84^), *Actinobacteria*, *Proteobacteria* and *Verrucomicrobia*.^84^, ^85^ The Firmicutes phylum is composed of over 200 genera including *Clostridium* (most abundant), *Lactobacillus*, *Enterococcus*, and *Ruminicoccus*. *Bacteroides* and *Prevotella* are the main genera within the Bacteroidetes phylum. *Bifidobacterium* genus is its most prevalent member of the Actinobacteria phylum. *Akkermansia muciniphila* (A. muciniphila) was thought to be the sole species within phylum Verrucomicrobiota until recently. Similar phyla than in humans dominate the rodent gut,^86^ and therefore rodents are used to model links between the gut microbiome and various diseases, including cancer. Three markers of a healthy gut microbial composition have emerged: (i) a high gut microbial alpha diversity, reflecting a presence of high number of different bacterial genera and species within a person's gut microbiota^87^, ^88^; (ii) high Firmicutes to Bacteroidetes (F/B) ratio^89^; and (iii) high levels of bacterial metabolites short chain fatty acids (SCFAs).^90^, ^91^, ^92^

### 3.2 Gut microbiota and breast cancer

Studies that have investigated if the microbiota is linked to breast cancer risk show a difference in the composition of the breast microbiota in normal breast tissue between healthy women and women with breast cancer,^93^ between breast cancer and breast tissue with benign growth,^94^ or between breast cancer and normal adjuvant breast tissue.^95^, ^96^ Consistent with this, the gut microbiota is different between individuals with cancer, including breast cancer, and those who are cancer free.^97^, ^98^ A recent study reported a lack of *Megamonas* and *Akkermansia* in the gut microbiota in metastatic breast cancer patients, compared with non‐metastatic patients.^99^ In the same study, it was also reported that the gut microbiota composition in metastatic breast cancer patients was predictive of changes in lipid transportation and metabolism and folate biosynthesis.^99^ However, these differences mostly reflect cancer‐induced changes in the microbiota rather than changes, which cause cancer.

In our unpublished study, the composition of the gut microbiota is dramatically altered after mice have been allografted mammary tumor cells and develop cancers, compared with the gut microbiota of the same mice before they received allografted tumor cells. Bacteria genera that were present at a significantly higher abundance in the fecal samples in mice with cancer included *Clostridium* sensu stricto, *Streptococcus* and *Turicibacter*. *Clostridium* sensu stricto is highly elevated in pancreatitis.^100^ *Streptococcus* is a well‐known pathogen and also a biomarker of several cancers.^101^ *Turicibacter* is increased when CD8+ T cells are ablated.^102^ These changes in the gut microbiota are likely to drive tumor growth, because if the gut microbiota is cleaned, tumors grow slower. However, we are aware of only one study that have investigated the causality between gut microbiota composition and breast cancer risk. In this preclinical study, fecal microbiota transplant (FMT) from mice at increased mammary cancer risk due to being fed OID to control mice increased mammary cancer in the control mice.^103^ These findings indicate that the composition of the gut microbiota may be related to breast cancer risk. In another preclinical study, gut dysbiosis was induced by antibiotic treatment. Mice with gut dysbiosis exhibited increased higher dissemination of mammary tumor cells to tumor‐draining lymph nodes and lungs than mice with intact gut microbiota.^104^

Gut microbiota also modifies responsiveness to cancer therapies.^105^ It has been investigated if the composition of the gut microbiota is linked to treatment with endocrine therapy^106^ or response to HER2 blocking monoclonal antibody trastuzumab.^107^ Aromatase inhibitor letrozole decreased the abundance of Bacteroidetes and increased SCFA producing Firmicutes, including *Lachnospiraceae* and *Ruminococcaceae* in a preclinical model.^106^ In a human study, FMT was obtained from HER2+ breast cancer patients exhibiting pathological complete response to neoadjuvant trastuzumab and from patients not responding to the treatment.^107^ Mice receiving FMT from responding patients also responded to trastuzumab, while mice receiving FMT from non‐responding patients did not. Trastuzumab responding patients exhibited a higher abundance of SCFA producing *Clostridiales* bacteria and lower abundance of *Bacteroidales*. Taken together, emerging evidence links gut microbiota composition to response to breast cancer therapies.

## 4 GUT MICROBIOTA AND RESPONSIVENESS TO ICBs

The evidence to indicate that gut microbiota composition is a critical player in determining the response to ICBs is multifaceted. It was first discovered that syngeneic mice with similar genetic background that were allografted the same mouse tumor cells exhibited significant differences in the response to ICBs based on the composition of their gut microbiota.^108^ Further, the responsiveness to ICBs was associated with activation of CD8+ T cells.^108^ Three human studies confirmed that indeed the gut microbiota was causally related to ICB response in non‐small cell lung cancer (NSCLC),^109^ and melanoma.^110^, ^111^ Although each of these studies identified a different gut microbiota signature linked to ICB response, the gut microbiota that favored response to ICB was associated with increased antigen processing and presentation, higher CD8+ T cells and lower Foxp3/T cells in the TME.

In a comprehensive review by Oh et al^112^ and another systemic review by Huang et al^113^ that assessed a connection between the human gut microbiota and responsiveness to immunotherapies, three markers of response to ICB emerged. These are (i) high alpha diversity, (ii) high abundance of Firmicutes, compared with Bacteroidetes, and (iii) high abundance of *Lachnospiraceae* and *Ruminococcaceae* families that are the main SCFA producers,^114^, ^115^, ^116^ and high levels of SCFAs. Of various bacterial species, *Bifidobacterium longum* or *adolescentis* or *Akkermansia muciniphila* were present in high abundance in melanoma, NSCLC, renal cell cancer and hepatocellular cancer patients responsive to ICBs. Similar to the initial three human studies, the studies performed after them and included to the two reviews indicated that activation of CD8+ T cells was seen in ICB responsive patients.^112^, ^113^

### 4.1 Obesity and gut microbiota

Obesity influences the gut microbiota, and the gut microbiota influences adiposity.^117^, ^118^, ^119^ However, different studies report different and sometimes opposing effects of obesity on specific changes in the gut microbiota. The effects of obesity on alpha diversity are an illustrating example. Alpha diversity has been reported to be increased in obese African Americans,^120^ not to be altered in obese non‐Hispanic Caucasian,^120^ and reduced in a large cohort of twins living in the United Kingdom.^121^ A recent systemic review and meta‐analysis show that obesity does not reliably affect alpha diversity.^122^ Obesity increases Firmicutes and reduces Bacteroidetes,^123^, ^124^ but in some studies, obesity reduced Firmicutes to Bacteroidetes ratio.^89^ Obese individuals exhibit increased fecal SCFA levels.^125^ The effects of obesity on the gut microbiota; that is, increased alpha diversity, F/B ratio and increased levels of SCFAs, might partly explain why obesity improves response to ICBs, as discussed above.

### 4.2 Fecal microbiota transplants

The most direct evidence indicating that the gut microbiota is determining ICB response originates from the FMT studies. FMT from ICB responding patients generates ICB response in mice, while FMT from ICB refractory patients fails to initiate ICB response.^109^, ^110^, ^111^ Similar results have been obtained in two recent studies in which PD1‐refractory melanoma patients received FMT from responsive patients, and consequently also started to respond to ICB.^126^, ^127^ The gut microbiota of patients receiving FMT and subsequently becoming ICB responsive was enriched in the taxa of the phylum Firmicutes, especially SCFA‐producing *Lachnospiraceae* and *Ruminococcaceae* families. The bacteria that were decreased in the responders were mostly Bacteroidetes. Importantly, FMT responding patients exhibited higher levels of activated CD8+ T cells with higher cytolytic functions than FMT non‐responders and the proportion of immunosuppressive myeloid‐derived suppressor cells (MDSC) was lower in the FMT responding patients,^127^ Thus, the gut microbiota seems to be involved in determining response or resistance to ICBs, potentially through mechanisms which alter the tumor immune microenvironment.

## 5 HOW GUT MICROBIOTA AFFECTS IMMUNE AND INFLAMMATORY RESPONSES

Gut microbiota and bacterial metabolites affect hematopoietic stem cell maturation in the bone marrow.^128^, ^129^ Gut microbiota/germ‐free (GM) mice, housed in germ‐free environment, exhibit reduced proportions and differentiation potential of myeloid progenitor cells of bone marrow origin, compared with specific‐pathogen‐free (SPF) mice. SPF mice are housed in pathogen‐free environment which is free from a selection of common pathogens, and they have normal but pathogen free gut microbiota. The impaired progenitor cells in the GM mice can be rescued with colonization of their gut microbiota with FMT from SPF mice.^130^ It was further reported that co‐housing SPF mice with mice raised in a conventional living environment improved their innate (CD11b+ cells) and acquired immunity, including the numbers of CD4+, CD8+ and memory T effector cells and B cells.^131^

The immune cells in the gut, other organs and tumor microenvironment can identify microbial metabolites via toll like receptors (TLRs) and other pattern recognition receptors (PRRs) in the immune cells. The bacterial products that impair immune cells include lipopolysaccharide (LPS).^132^, ^133^, ^134^, ^135^ LPS is a metabolite excreted by microbes of the Bacteroidetes phylum, and it can promote tumor growth through activation of TLR4 signaling.^136^, ^137^ LPS/TLR4 signaling enhances proinflammatory cytokine production, cancer stemness capacity and proliferation of hepatic progenitor cells during hepatocarcinogenesis and hepatocellular carcinoma recurrence.^138^ LPS/TLR4 also increases Foxp3 expression and immunosuppressive capacity of Treg cells *in vitro*.^139^ Further, TLR4 activates immunosuppressive and pro‐carcinogenic pathways such as the nuclear factor (NF)‐κB and JAK/STAT3 signaling pathways.^140^ SCFAs and immune responses are discussed below.

Finally, gut microbiota metabolites can impact immune responses via extracellular vesicles (EVs). Gut bacteria derived EVs can disseminate to distant organs and TME delivering proteins, enzymes (such as autolysins) and toxins, polysaccharides, nucleic acids (DNA and RNA), and peptidoglycan. Any of the EV cargo may then impact local immunosurveillance.^141^ Whether gut microbiota derived EVs suppress or promote tumor immune effector cells depends on whether they originate from pathogenic or commensal bacteria.^142^, ^143^

### 5.1 The gut microbiota‐immune response connections: Short chain fatty acids

As mentioned above, high SCFAs are seen in healthy individuals, and they are linked to response to HER2 inhibition and ICB responsiveness in cancer. In addition, SCFAs are considered as an important link between the gut microbiome and tumor immune response.^144^, ^145^ SCFAs are produced by certain bacteria when dietary fiber or carbohydrates are consumed.^146^ SCFAs have a chain length of up to six carbons atoms, and most of them are acetate, propionate and butyrate. The gut bacteria which generate most of the butyrate are Firmicutes, in particular those of the families *Ruminococcaceae* and *Lachnospiraceae*.^115^, ^116^ *A. muciniphila* produces both propionate and acetate^116^, ^147^ and *Bifidobacteria* produces acetate.^148^ *Prevotella* of the Bacteroidetes phylum has been reported to produce^149^ or inhibit the production of acetate in the gut.^150^

Since fecal SCFAs can be promptly absorbed in the colon,^151^, ^152^ they likely impact both local and systemic immune responses.^90^, ^91^, ^153^ SCFAs bind and activate their G protein receptors GPR41, GPR43, and GPR109, olfactory receptor 78 (OLF78), or they are taken into a cell by sodium‐coupled monocarboxylate transporters, such as SLC5A8. Of the SCFA receptors, GPR43 and GPR109A are expressed in immune cells and have anti‐inflammatory properties.^154^ They also are expressed in the intestinal epithelium and the adipose tissue.^155^ GPR41 also is expressed in intestinal epithelial cells as well as sympathetic ganglia, and this receptor regulates glucose metabolism, appetite, heart rate and energy expenditure.^146^ OLF78 regulates blood pressure and is expressed in renal blood vessels.^156^ Through the different receptor types, SCFAs have widespread effects on different physiological functions, including enhancing mitochondrial functions and fatty acid metabolism.^157^

SCFAs has multiple effects on the immune cells, either through their receptors, specific transporters or acting as histone deacetylase (HDAC) inhibitors to activate epigenetically silenced immune genes.^92^, ^158^, ^159^ Butyrate and propionate modulate CD4+ T cell differentiation to Foxp3+ IL10‐producing Treg cells through cytokine production in the colon and extrathymic organs.^160^, ^161^ The effects of butyrate on Treg cell expansion depend on the butyrate concentrations and TGF‐β1. In high concentrations (>1 mM *in vitro*) or in the absence of TGF‐β1, butyrate induces expression of T‐bet and interferon γ (IFNγ) on Treg cells which then inhibit the differentiation of Treg cells.^162^ SCFAs can alter hematopoiesis by enhancing the generation of macrophage and dendritic cell (DC) precursors.^128^ Consequently, SCFAs may induce Treg cell differentiation through activation of dendritic cells and macrophages.^163^, ^164^ Through HDAC inhibition, butyrate either induce Treg differentiation by promoting Foxp3 protein acetylation, or by endowering dendritic cells with ability to facilitate Treg cell differentiation.^165^ In CD8+ T cells, butyrate and acetate systemically induce expression of IFNγ and granzyme B (GrB) through induction of histone acetylation.^166^ Expression of IFNγ and GrB in anti‐tumor CD8+ T cells is indicative of activation of these cells.

### 5.2 Causal link between gut microbiota, immune responses and cancer

In many human and preclinical studies that show a causal association between gut microbiota and response to ICBs, activation of CD8+ T cells is connected to the ICB response improving gut microbiota. Direct evidence linking the gut microbiota to cancer via immune responses can be obtained from studies showing that depletion of gut microbiota in syngeneic mice decreased the growth of pancreatic and colon cancer and melanoma as well as reduced their metastasis, but did not alter the tumor burden in Rag‐1 knockout mice which lack mature T and B cells.^134^ The findings obtained in Rag‐1 mice indicating no effects on tumor growth by gut microbiota cleaning suggest that the gut microbiota of tumor bearing mice contains bacteria, which promote cancer growth via modifying activation of adaptive immune system. It has also been shown that gut dysbiosis decreases the intestinal expression of IL4 and TGFβ, and IL9 producing T cells which have potent antitumor effects.^167^ Similar changes have been reported in the lung TME.^167^

## 6 GUT MICROBIOTA AND RESPONSE TO IMMUNOTHERAPY IN OFFSPRING OF OBESE MOTHERS

### 6.1 Short chain fatty acids

Maternal obesity is associated with lower levels of SCFA producing bacteria in the offspring's gut microbiota.^168^ For example, abundance of acetate producing *Bifidobacteria* was reduced in the OID offspring in two studies.^169^, ^170^ In preclinical and human studies, maternal obesity reduces the abundance of anti‐inflammatory, SCFA‐producing *Clostridiaceae* in the offspring.^170^, ^171^ Maternal obesity also increases the genus of *Prevotella* among offspring in humans and non‐humans primates^172^, ^173^, ^174^ and in rats.^175^ *Prevotella* inhibits SCFAs.^150^, ^176^ Prevotella also increases the release of cytokines and chemokines, and is therefore suggested to promote chronic inflammation and increase the risk of diseases in which inflammation plays an important role, including cancer.^177^

Consistent with the observed changes in the SCFA‐producing bacteria, maternal obesity is linked to reduced levels of SCFAs. Studies exploring changes in SCFA levels in obese mothers or their offspring have found that propionate levels in the circulation were reduced in obese rat dams, but no changes in acetate or butyrate levels were observed.^178^ In a study in mice, maternal obesity reduced butyrate levels in the dams^179^; no changes in other SCFAs were noted. Among the offspring, maternal obesity reduced acetate and propionate, but not butyrate levels in fecal samples of mice.^168^ A human study suggested that offspring of obese mothers had lower SCFA levels.^180^ Considering the evidence that high levels of gut microbiota species that produce SCFA may improve response to PD1 inhibitors,^181^ maternal obesity could program the offspring to resistance to ICB therapy.

SCFAs produced by the maternal gut bacteria during pregnancy are sensed by SCFA receptors GPR41 and GPR43 in the embryos; these receptors influence development of an embryo's metabolic and neural systems.^182^ Maternal SCFAs likely also program the immune system in the offspring.^183^, ^184^ Importantly, the lack of SCFA receptors in knockout mice has been shown to impact the composition of their gut microbiota,^185^, ^186^ possibly explaining how maternal obesity can determine the composition of offspring's gut microbiota.

### 6.2 Maternal obesity will permanently alter the composition of offspring's gut microbiota: Alpha diversity

In mice, maternal obesity or intake of obesity‐inducing high fat diet (OID) has been reported to either increase^187^ or reduce alpha microbial diversity^63^ among the offspring. Recent meta‐analysis of human studies also indicated that microbial diversity was either reduced or increased in the offspring of obese mothers.^173^ It is possible that the diversity is reduced in OID offspring that have not yet reached puberty, but later the diversity in these offspring is increased.^63^ We found increased gut microbial diversity in 8‐month‐old rats born to dams fed OID during pregnancy.^175^ In humans, increased bacterial diversity was seen in 6‐week‐old infants of obese mothers born vaginally but not via c‐section.^188^ Increased microbial diversity also has been reported in 18–27‐month‐old toddlers of obese mothers who had high socioeconomic status.^172^ Stanislawski^170^ found reduced microbial diversity in obese mothers, but no change in their offspring who were followed for up to 2 years of age.

### 6.3 Firmicutes to Bacteroidetes ratio

Another finding that differs among the studies involving offspring is whether maternal obesity increases or reduces Bacteroidetes phylum, and the F/B ratio. In our study, maternal obesity increased Bacteroidetes and reduced F/B ratio in OID offspring.^175^ In non‐human primates, Bacteroidetes were present in a higher abundance in the offspring of obese than lean mothers.^174^ Human studies have reported both an increase and a reduction in Bacteroidetes in infants born to obese mothers. A systemic review of 11 eligible human studies (out of 249 articles the investigators assessed)^173^ identified only six studies that had collected fecal samples from children of obese mothers or mothers exhibiting excessive pregnancy weight gain.^169^, ^170^, ^172^, ^189^, ^190^, ^191^ In offspring of obese mothers, the abundance of Bacteroidetes was increased in those born via vaginal delivery^189^ or to a high‐income family,^172^ while their abundance was reduced in infants born via c‐section^189^ or to a low‐income family of obese mothers.^172^ All the contrasting findings regarding F/B ratio that is a marker of ICB responsiveness make it challenging to predict if maternal obesity increases or reduces refractoriness to immune therapies.

**FIGURE 1:**
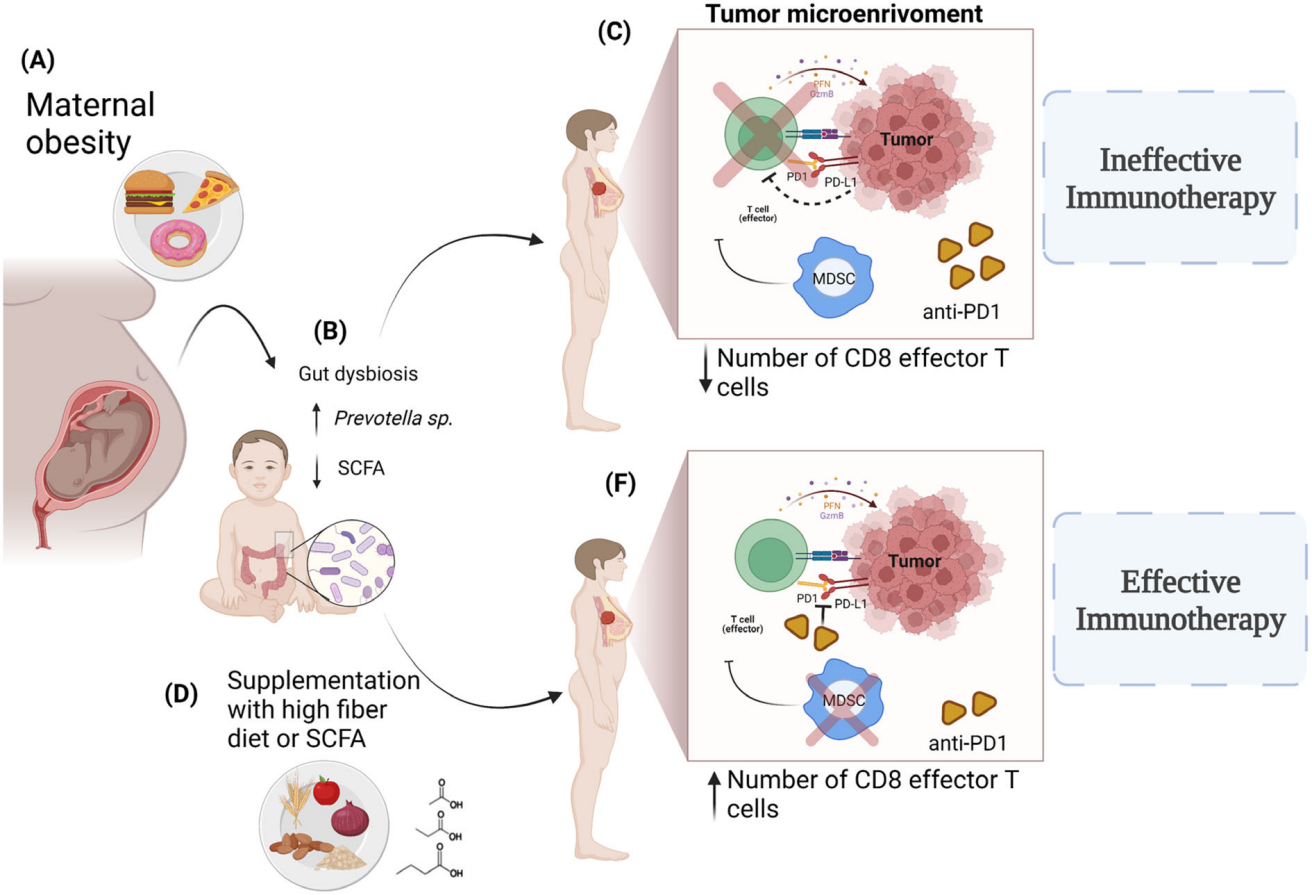
(A) Maternal obesity leads to gut dysbiosis in the offspring, manifested as higher levels of *Prevotella* sp. and reduced abundance of bacteria producing short chain fatty acid (SCFA) and consequent reduced levels of fecal and circulating SCFAs (B). High abundance of *Prevotella* sp. and reduced levels of SCFAs affect the tumor immune response prompting ineffective immunotherapy. Due to reduced number of CD8+ T cells, anti‐PD1 treatment cannot activate a sufficient numbers of CD8+ T cells and consequently does not inhibit tumor growth. Further, maternal obesity also increases the frequency of immunosuppressive cells, such as myeloid‐derived suppressor cells (MDSC) in the tumor microenvironment (TME) (C). Dietary interventions that increase the abundance of bacteria producing SCFAs and decrease *Prevotella*, such as supplementation with a high fiber diet, potentially reverses offspring's resistance to breast cancer immunotherapy through increasing CD8+ T cells (D). In this condition, anti‐PD1 treatment will induce tumor immune response preventing CD8+ T cells exhaustion (F). Created with BioRender.com

To summarize, the gut microbiota may be particularly important in the context of maternal obesity and offspring's mammary tumorigenesis and resistance to immunotherapy for the following reasons: (i) the composition of the gut microbiota, which is unique to each individual, is established early in life, and is then maintained relatively stable throughout the life,^192^, ^193^ (ii) maternal obesity induces persistent gut dysbiosis among offspring,^63^, ^174^, ^187^, ^191^, ^194^, ^195^ and (iii) gut dysbiosis is linked to increased cancer and resistance to immunotherapy.^104^, ^196^ Further, (iv) the gut microbiome can modify the epigenome of immune cells,^197^, ^198^ for example by altering the levels of SCFAs that are produced by certain bacteria when dietary fiber or carbohydrates are consumed.^146^ Figure 1 summarizes the connection between maternal obesity and offspring's potential resistance to ICBs.

## 7 IMPLICATIONS ON OFFSPRING'S HEALTHY OF CHANGING THE GUT MICROBIOME IN DAMS USING PROBIOTIC OR PREBIOTIC SUPPLEMENTS

### 7.1 Probiotic and prebiotic supplementation of pregnant mothers

Emerging literature describes the effects of various prebiotic and probiotic supplements on the gut microbiome and health.^199^, ^200^ Probiotic supplements contain a single or multiple bacterial strain. Prebiotic supplements originate from fiber and carbohydrates, and the most common of them are fructo‐oligosaccharides (FOS), galacto‐oligosaccharides (GOS), and trans‐galacto‐oligosaccharides (TOS). When prebiotics are fermented by the gut microbiota, the gut produces SCFAs. Several studies have investigated if the adverse effects of maternal obesity on offspring can be reversed by modifying the gut microbiota with probiotic or prebiotic supplements, either given to pregnant mothers or to their offspring. When assessing individual studies, maternal supplementation with *Lactobacillus and/or Bifidobacterium* controlled pregnancy weight gain among obese mothers^201^ and reduced an offspring's risk of developing eczema in humans.^202^ In mice, supplementation of OID fed dams with *Lactobacillus* and *Bifidobacterium* reduced metabolic abnormalities and reversed gut dysbiosis among the offspring.^194^ Supplementation of OID fed rat dams during pregnancy with *Lactobacillus* prevented hypertension in their offspring.^203^

However, a systemic review with meta‐analysis evaluated if maternal probiotic supplement use affected outcome of pregnancy.^204^ For that purpose, 1441 publications were screened and 76 of them were included to the analysis. Although maternal probiotic supplementation initially altered offspring's gut microbiota composition, no persistent effects on the infant gut microbiome were observed.^204^ Another systematic review and meta‐analysis also did not find any evidence that maternal probiotic supplementation beneficially impacted the potential adverse maternal and infant outcomes, including gestational diabetes, preterm birth, or small‐ and large‐for‐gestational age.^205^

### 7.2 Probiotic supplementation of offspring of obese pregnant mothers

Since most obese women did not take prebiotic or probiotic supplements during pregnancy, it is important to determine if supplementation of OID offspring is able to reverse the adverse effects caused by maternal obesity. Cognitive and social deficits in the OID rat offspring can be prevented by supplementing offspring with *Lactobacillus*.^206^ In other studies in rats^207^ or primates,^208^ supplementation of adult OID offspring with probiotics alone or with both prebiotics and probiotics had beneficial effects on offspring's gut microbiota and metabolic profile, including lowering triglycerides and cholesterol levels. These results suggest that probiotics may be more potent in improving health of OID offspring, if they are given directly to offspring rather than pregnant obese mothers.

However, although probiotics may have beneficial effects if used by offspring of obese mothers, it is important to point out that the ability of probiotics to alter the gut microbiota remains unclear.^209^ For example, an exposure of germ‐free mice to an 11‐strain probiotic mix effectively colonized their gut mucosa, while colonized microbiota in conventionally housed mice exhibited a marked resistance to probiotics.^210^ In humans, response to probiotics is highly individualized.^210^ Further, probiotics reduce the diversity of the microbiota.^211^ Since higher microbial diversity may protect against cancer and resistance to ICB therapy,^112^, ^209^ probiotics may not be an ideal way to improve the composition of the gut microbiome to improve health or the response to cancer therapies.

### 7.3 Prebiotics

Prebiotics may be better for cancer prevention in the offspring of obese mothers, and even improving responsiveness to anti‐PD1 cancer therapy.^212^ A recent study compared the effectiveness of two soluble dietary fibers—inulin and mucin—against colon cancer and melanoma in C57BL/6 mice.^213^ This study did not involve obese dams, but the supplementation was directed to adult mice with allografted tumor. Inulin is a naturally occurring fructosyl polymer with chain‐terminating glucosyl residues. Its dietary sources include garlic, onions, leaks, rye, barley, banana and many root vegetables. Mucins are highly decorated with polysaccharides composed of various core structures similar to those found in Lewis blood type antigens, including various sugars. Mucins are present for example in human and bovine milk. Inulin and mucin both inhibited melanoma tumor cell growth in syngeneic mice, and when given in combination, they were more effective than either prebiotic alone. Only inulin inhibited colon cancer. Both prebiotics affected tumor immune responses. Inulin activated CD4+ and CD8+ T cells, while mucin stimulated antigen presentation. The two prebiotics had both similar and different effects on the gut microbiota. The differences were in the bacteria they altered, while the similarity was the gut metabolite that was affected. Among the phylotypes induced by inulin and mucin were taxa in the SCFA butyrate‐producing bacteria. As discussed above, SCFA producing bacteria have been linked to several anti‐cancer mechanisms and improve response to immunotherapy.^90^, ^214^, ^215^

The effects of soluble fibers, when given to pregnant dams, on offspring's immune cells has been investigated. Two studies showed that prebiotic supplementation with galacto‐ and fructo‐oligosaccharides + inulin led to tolerogenic immune imprinting in the offspring.^216^, ^217^ The supplementation also prevented development of wheat allergies in the offspring.^217^ In another mouse study, polydextrose supplementation of obese pregnant mice improved maternal glucose homeostasis and prevented offspring from becoming obese.^218^ Hsu et al^219^ exposed pregnant mice to high fructose diet that causes hypertension in the offspring. Hypertension was prevented by supplementing pregnant dams with *Lactobacillus casei* or inulin. In addition, both treatments increased the abundance of *Akkermansia muciniphila* and reduced *Prevotella albensis* in the offspring. Since high abundance of *Akkermansia muciniphila* and reduced *Prevotella* is linked to better response to immunotherapy,^112^ supplementation with *Lactobacillus casei* or inulin may reverse the adverse effects of maternal obesity in the offspring's gut microbiota, with consequently better outcome with ICB therapy.

Finally, since maternal obesity suppresses SCFAs, it was investigated if supplementing OID offspring with SCFAs acetate and propionate might prevent the adverse effects of maternal obesity in offspring: *it did*.^220^ In another study, dams fed OID, or their offspring, were supplemented with inulin, and inulin supplementation reversed reduction in SCFAs both in the dams and offspring.^168^ In addition, inulin reversed cognitive and social defects in the OID offspring. Considering that high fiber diet induced changes in the gut microbiota, leading to increased production of SCFA increases CD8+ T cell activation and enhance memory potential of activated CD8+ T cell,^128^, ^221^ these data suggest that supplementation with dietary fiber diet or SCFAs may improve response to immunotherapy among offspring of obese mothers.

## 8 CONCLUSIONS

Maternal overweight and obesity casts a dark shadow on the health of the next generation by increasing their risk of developing diseases that are considered curses of the modern civilization, such as Alzheimer's disease,^222^, ^223^ autism,^224^ cardiovascular diseases,^225^, ^226^, ^227^ obesity and type 2 diabetes^228^, ^229^, ^230^, ^231^ as well as many cancers, including breast cancer. Maternal obesity also may impair the offspring's ability to respond adequately to treatments of these diseases. Several mechanisms have been proposed to mediate the adverse effects of maternal obesity, including increased inflammatory environment.^57^, ^232^ The gut microbiota may be particularly important as a mediator of the effects of maternal obesity on the offspring, as maternal obesity induces gut dysbiosis among offspring.^63^, ^174^, ^194^, ^195^ Gut dysbiosis is linked to Alzheimer's disease,^224^, ^233^ autism,^234^, ^235^ cardiovascular diseases,^236^, ^237^, ^238^ obesity,^239^, ^240^, ^241^, ^242^, ^243^ and type 2 diabetes.^222^, ^244^, ^245^ Thus, efforts should be focused on identifying means to prevent gut dysbiosis in the children born to obese mothers.

No specific bacterial family, genus or species has emerged as a marker and potential mediator of the effects of maternal obesity on offspring. The strongest candidate is increased abundance of Bacteroidetes, especially *Prevotella*.^173^, ^175^ If confirmed to be true, attempts need to be directed to suppress *Prevotella* instead to the current trend to supplement with various bacterial species (mostly of *Bifidobacterium* or *Lactobacillus* genera or *Akkermansia muciniphila*). We also believe that supplementation with prebiotics/dietary fiber is a more effective approach than probiotic supplementation, especially during pregnancy, because prebiotics alter the abundance of many bacteria and likely in a manner that considers unique inter‐individual differences in the composition of the gut microbiota. Further, it is possible that the critical factor in determining the effect of maternal obesity on offspring, and offspring's gut microbiota then increasing a risk of developing different diseases, is the metabolites the gut microbiota produces. For example, maternal obesity suppresses SCFAs in the offspring,^168^ and SCFAs are critical for normal immune responses, including anti‐tumor immunity.^90^, ^91^, ^153^ Supplementation of pregnant mother with dietary fiber inulin that elevates fecal SCFA levels in the mother and her offspring has been reported to reverse some adverse long‐term health effects of maternal obesity.^168^ More research is urgently needed to determine if offspring can be rescued from increased disease burden by simple and safe dietary modifications taking place either during pregnancy or in offspring.

Since immunotherapies have emerged as highly effective treatments for many cancers, albeit there is an urgent need to enlarge the patient population who will be responsive to these treatments by identifying the key factors which determine ICB responsiveness. One of the factors which promote ICB refractoriness may be maternal obesity, based on its effects on the microbiota markers of ICB therapy response. Based on the vast numbers of children born to obese mothers in the modern society, it is important to carry out studies to assess whether maternal obesity impairs offspring's response to cancer immunotherapies.

## AUTHOR CONTRIBUTIONS

**Fabia de Oliveira Andrade:** Conceptualization (equal); visualization (lead); writing – original draft (equal); writing – review and editing (equal). **Vivek Verma:** Conceptualization (equal); visualization (supporting); writing – original draft (supporting); writing – review and editing (equal). **Leena Hilakivi‐Clarke:** Conceptualization (equal); funding acquisition (lead); project administration (lead); visualization (supporting); writing – original draft (equal); writing – review and editing (equal).

## CONFLICT OF INTEREST

The authors declare no conflict of interest.

### ETHICS STATEMENT

Authors declare that the work in the present manuscript is in accordance with the Publication Ethics Guidelines as laid down by Committee on Publication Ethics. It is further declared that no part of the present manuscript has been published or is under consideration in other journal.

## Data Availability

N/A.
